# Health Benefits of Social Media Use in Adolescents and Young Adults

**DOI:** 10.1007/s40124-025-00357-7

**Published:** 2025-08-15

**Authors:** Jason M. Nagata, Oliver Huang, Jacqueline O. Hur, Elizabeth J. Li, Christiane K. Helmer, Emily Weinstein, Megan A. Moreno

**Affiliations:** 1 Department of Pediatrics, University of California, San Francisco, 550 16th Street, 4th Floor, Box 0503, San Francisco, CA 94143 USA; 2 Center for Digital Thriving, Harvard Graduate School of Education, 13 Appian Way, Cambridge, MA 02138 USA; 3 Department of Pediatrics, University of Wisconsin, 500 Lincoln Dr, Madison, WI 53706 USA

**Keywords:** Social Media, Adolescence, Health Education, Social Connection, Identity Development, Mental Health

## Abstract

**Purpose of Review:**

Although attention has increased on the negative aspects of social media use in adolescents and young adults, social media can have health benefits. This review explores positive health aspects of social media and delivers guidance to clinicians on how to balance attention to the negatives and positives of social media use.

**Recent Findings:**

Recent findings show social media can play an important role in social connection and identity development in adolescents and young adults. The platforms are also important for mental health support and accessing health information.

**Summary:**

Clinicians should have open-ended conversations with adolescents and young adults to understand their social media use patterns. They can use the American Academy of Pediatrics 5 Cs of media use and the family media plan to guide conversations on social media use.

## Introduction

Social media use is ubiquitous in adolescent life. Among US 13–17-year-olds, 90% use YouTube, 63% use TikTok, and 61% of teens use Instagram [1]. Nearly two-thirds (64%) of 11–12 year olds report social media use, and 11–12 year old users have on average 3.4 social media accounts [2]. Social media use also increases as adolescents get older. From ages 9 to 13, daily time on social media increases from 7 min a day to 73 min a day [3].

Although social media use can have both risks and benefits, past research has largely emphasized the negative consequences of social media use in adolescents. Adolescent social media use has been associated with high rates of depression, anxiety, and eating disorders [4, 5]. Adolescent social media use is also linked to higher likelihoods of substance use and worsened sleep [6, 7]. However, there is evidence that problematic social media use, characterized by symptoms including sleep disturbances, risky use, compulsive use, or addiction, may mediate these negative associations, calling into question whether social media itself is driving these relationships or unhealthy engagement patterns [4].

Parents are increasingly concerned about their adolescents’ social media use. Screen time and social media are the top two health concerns of parents [8]. The Surgeon General’s advisory on parenting mental health highlighted technology and social media as the top two reasons parenting is more difficult than it was 20 years ago [9]. 70% of parents are somewhat, very, or extremely worried that teens are wasting too much time on social media [10].

To address the issue of social media and technology, pediatricians must communicate with adolescents about responsible social media use and assess the different risks and/or benefits that their adolescent patients encounter. Past studies have shown that adolescents prefer pediatricians who listen to their concerns and experiences [11]. When discussing sensitive subjects, such as sexual health and substance use, adolescents also prefer physicians with a nonjudgmental attitude [12]. The values of listening and nonjudgment can be applied when discussing social media use with adolescents. To do so, pediatricians can utilize the American Academy of Pediatrics 5 Cs framework. The framework asks physicians to consider the Child, Content, Calm, Crowding Out, and Communication when evaluating media use in adolescents and families [13]. Child refers to taking a child-centered approach when thinking about their reasons for using social media, and Content refers to what children are viewing [13]. Calm refers to how screen use may be used as a coping mechanism for emotions or for trying to fall asleep [13]. Crowding Out is about what is displaced by digital media use, and whether adolescents are crowding out activities such as sleep, physical activity or in-person connection [13]. Communication refers to communication between caretakers and adolescents about digital media use [13].

This review aims to fill literature gaps by exploring recent evidence on the potential benefits for adolescents that can come from social media use. It focuses on current ways adolescents utilize and experience social media, while excluding experimental interventions on adolescent social media use (e.g., adult-designed interventions using social media as a platform to reach adolescents). The review can inform discussion points to begin conversations with adolescents and their parents regarding social media use, acknowledging potential benefits adolescents may experience. The review may also allow clinicians to tailor individualized social media guidance to parents and adolescents to maximize the benefits of social media use while mitigating the harms.

### Improved Mental Health through Social Connection

Social media can help adolescents maintain and build new social connections with peers. Focus groups report that teens use social media to keep in touch with peers from primary school. For teens whose classmates live far apart, teens describe social media as an important way to keep in touch [14]. Teens specifically describe group chats as fostering a sense of belonging [14]. Similarly, adolescent girls who spend more time with close friends on WhatsApp and Instagram reported stronger friendship closeness [15]. Adolescents specifically identified Instagram as a key platform for maintaining and expanding new friendships [16]. Broader studies support these findings, showing a positive correlation between social media use and feelings of connectedness [17]. Notably, the type of engagement matters: active participation on social media, such as engaging with posts, is associated with reduced loneliness, while passive use, such as doomscrolling, does not show the same effect [18].

Social media helped adolescents stay socially connected during COVID-19 pandemic lockdowns [19], which limited face-to-face interaction. Teens reported using social media to keep in touch with classmates and friends [20]. During the COVID-19 pandemic, video chats, which can occur on social media platforms, were tied to feeling more connected and less lonely [21, 22], and were even associated with lower depressive symptoms, unlike other forms of communication such as social gaming and voice calls [22]. Additionally, adolescents frequently turned to social media to help cope with pandemic-related anxiety [23]. Humorous content, particularly, helped adolescents cope with stress during the pandemic [19].

Similar patterns have been observed in adolescents hospitalized for mental health treatment. Hospitalized adolescents could utilize social media to maintain relationships with classmates, friends, and family [24]. Teens reported that maintaining relationships with friends could offer emotional support during hospitalization [25]. Beyond maintaining relationships, these adolescents also turn to social media as a means of emotional distraction from negative thoughts [25]. This suggests that social media can help foster social connection for adolescents hospitalized with mental illness.

### Identity Formation, Support, and Acceptance

For marginalized adolescents, social media offers a way to connect with other peers who share similar identities [26–28]. Sexual minority adolescents are more likely than heterosexual adolescents to seek community and support through online groups [29]. In interviews, these teens describe how social media enables them to connect with peers, offer mental health support, and express their identity [30, 31]. Surveys of sexual minority adolescents found that they use social media for emotional support [32]. LGBTQ + youth also feel more comfortable expressing their identity online compared to in person [28]. Consistent with these findings, a large-scale survey found that social media use is linked to higher feelings of social capital among sexual minority adolescents [33]. Sexual and gender minority adolescents who feel safe online were also less likely to experience symptoms of anxiety and attempt suicide [34].

Social media also serves as an important platform for racial and religious minority adolescents to build community. A majority of youth see comments affirming different racial and ethnic identities on social media [35]. Black and Hispanic teens are more likely than White teens to view social media as a key space for creative expression and connection [36–38]. Latino teens report that social media offers a way to connect with others in their community [37]. Focus groups with Muslim adolescents in Australia revealed that these platforms enable them to stay connected with communities, including nearby friends and relatives abroad [39].

Similarly, adolescents with chronic and rare diseases utilize social media to connect with others online and combat isolation. Those with Hypermobile Ehlers-Danlos Syndrome report using social media to befriend others with their condition and to better understand their condition [40], while adolescents with Sarcoma report using social media to maintain preexisting friendships [41]. Patients with Type 1 diabetes report using social media to connect with other adolescents with their disease. Teens with diabetes say social media helped accept their diagnosis and normalized their condition [42].

Social media may also promote acceptance of marginalized groups. In one experiment, when individuals were exposed to Facebook profiles of either transgender, schizophrenic, or disabled users, they had more accepting attitudes toward those groups [43].

### Mental Health Destigmatization and Support

Adolescents and young adults often disclose mental health struggles online, offering an avenue to identify mental health struggles in adolescents. Analyses of Facebook and WeChat posts indicate adolescents often share their mental health symptoms on these platforms [44, 45]. Similarly, adolescents with depressive symptoms are more likely to disclose negative emotions online [46]. This suggests that social media could serve as a useful tool for healthcare providers to identify or diagnose depression in adolescents.

Celebrity disclosures of mental health challenges can play a crucial role in reducing stigma. One survey found that celebrity discussions about depression may foster greater acceptance and spark more open conversations about mental health [47]. Additionally, viewers are more likely to sympathize with celebrities who they see as similar to themselves, which can further normalize mental health discussions [48].

Adolescents may turn to social media to access information and support when facing mental health challenges [49]. Semi-structured interviews with teens reveal that they turn to TikTok for coping strategies and a safe environment to discuss their mental health experiences. Teens reported that the app also encouraged them to seek mental health help [50]. Rural youth experience greater online social support compared to urban youth [51]. Many of the videos shared on TikTok feature personal experiences from therapists who have treated patients or teens who have experienced mental health struggles firsthand [52]. This underscores the role of social media in offering valuable emotional support to teens. Importantly, although teens viewed TikTok as an important tool for mental health help, most of them did not view the platform as a replacement for professional mental health help [50].

### Health Education Benefits

Social media has become a popular tool for adolescents seeking health information. While YouTube is the most popular platform for health information, teens also turn to other platforms such as Facebook and Instagram for health education [53]. In addition to gathering information, adolescents often post about their own health and share health-related content with others on these platforms [54].

Throughout the COVID-19 pandemic, social media has served as an important channel for experts to communicate public health information and share updates [55]. Adolescents who spent an hour or more a day on social media were more likely to consistently wear masks, indicating a potential role of these platforms in successfully conveying public health messages [56].

Despite concerns about its role in promoting unhealthy eating habits, social media may also serve as a powerful tool for promoting healthy behaviors among adolescents. Instagram and Facebook have been highlighted as key platforms that promote healthy eating in adolescents [57]. A content analysis of adolescent social media content on Instagram revealed a positive portrayal of fruits and vegetables [58]. Furthermore, qualitative interviews suggest that participation in social media-based fitness communities can motivate adolescents to adopt and maintain healthy lifestyles [59]. TikTok, in particular, is preferred by adolescents seeking information on healthy lifestyles and weight management [60]. Adolescents also prefer to use TikTok and Instagram to find social connection with others on weight loss and healthy lifestyles [60].

Adolescents and young adults may seek sexual and reproductive health information through social media, particularly when societal taboos create barriers to accessing this knowledge elsewhere. Peer-generated content on TikTok includes discussions about birth control, abortion, and miscarriage [61]. Research indicates that adolescents who engage with this content demonstrate higher levels of knowledge regarding contraception and HIV/AIDS [62], and racial minority youth who are exposed to social media information on sexual health were more likely to adopt protective behaviors, such as using condoms and other forms of contraception [63].

Although social media contains many health-related resources for adolescents, content accuracy may be a concern. An analysis of nutrition-related Instagram posts found nearly half of all posts contained inaccuracies [64]. Similarly, online content involving reproductive health frequently included misleading risks on medical procedures and recommendations that did not align with professional guidelines [65]. While health misinformation may be a problem, adolescents are often discerning about social media health content [66]. They often remain skeptical of sponsored content, and some adolescents independently fact-check health information they learn from social media [66].

### Clinical Implications

Social media use can have important benefits for adolescents. The American Psychological Association acknowledges the importance of social media use for health education [67]. Physicians can support families to adopt a nuanced and individualized approach when counseling adolescents and their families about social media use, utilizing the 5 Cs: Child, Content, Calm, Crowding Out, and Communication [13] (Table 1).

**Table 1:** Applying the 5 Cs to discussions on social media with adolescents

5 Cs of media use	Application to practice
Child—Understanding what drives children to use social media	Asking adolescents about their motivations to use social media, such as to find community online or find entertainment
Content—Understanding what content children watch on social media	Asking adolescents what content they consume on social media and explaining to adolescents how screen time quality can vary
Calm—Understanding how children fall asleep at night	Asking adolescents about bedtime screen use and identifying other ways to fall asleep
Crowding Out—Understanding how media use impacts the ability to engage in other activities	Working with adolescents to understand how screen time crowds out other activities and finding non-screen activities to replace screen time
Communication—Maintaining communication with adolescents	Encouraging periodic discussions with caregivers and adolescents on screen use

Physicians should understand adolescent motivations to use social media and their consumption habits on social media. These make up the Child and Content of the 5 Cs. Physicians should ask adolescents about their motivations for using social media, such as finding a community online or seeking entertainment. They should also ask adolescents what content they consume on social media and explain to adolescents how screen time quality can vary. For marginalized youth, who may turn to social media for identity affirmation and community-building, counseling should address both the value of online community and the need for safety and privacy online. Additionally, given adolescents’ use of social media as a health information source, physicians should also emphasize the importance of evaluating the accuracy of information and advice accessed through social media platforms.

Physicians should work to find balance online and offline time. The Calm and Crowding Out of the 5 Cs emphasize the importance of responsible screen use. Although social media use may help youth find community, physicians should ask adolescents about bedtime screen use and identifying other ways to fall asleep. To reduce screen use, physicians should work with adolescents to understand how screen time interrupts other activities and find non-screen activities to replace screen time.

Finally, physicians should encourage adolescents and parents to discuss social media use on a regular basis (Table 1). Past studies indicate encouragement by pediatricians was associated with increased reported communication between adolescents and parents [68]. Topics between adolescents and parents can include the content adolescents consume, the frequency content is consumed, and boundaries. For example, while adolescents may come across health information on social media, this information may be inaccurate. Discussions between parents and adolescents can foster better relationships regarding screen and social media use, and they can teach adolescents to use social media more responsibly.

One way to encourage these discussions is to encourage parents and children to set up a family media plan, which includes individualized guidance for each household. The family media plan acknowledges there is not a one-size-fits-all solution for screen and social media rules, and can be personalized based on children’s ages, what electronic devices are in the household, and the family’s needs for communication and schoolwork. An adapted version of the family media plan specific to adolescent social media use is shown in Table 2.

**Table 2:** Family media plan components adapted to adolescent social media use

Social media balance	Communicating with social media	Digital privacy and safety	Social media free zones and times	Choosing good content
Tracking social media use	Talking about the ways social media can affect mental health	Trying to avoid oversharing	Keeping meals social media free	Being more intentional about content consumed on social media
Participating in other activities in our community	Knowing when and how social media is inappropriate or harmful	Avoiding social media land mines, rabbit holes, and inappropriate content	Keeping bedrooms social media free at night	Prioritizing creative, educational, prosocial, and positive content
Having fewer social media apps on our devices	Communicating with adults about online harassment and bullying	Exploring privacy settings and setting them at the highest level of security	Holding off on social media use until age 13 or older, with parent support	Making a plan for spending money online
Setting time limits on social media and ensuring use doesn't interfere with other activities	Respecting others on social media	Setting and following safety rules for chatting on social media	Having a plan for devices at school	
Realizing when we turn to social media to dull our own emotions, and finding healthier ways to cope			Choosing which days of the week are OK for social media	
Playing apps and games or watching videos together as a family			Having a homework-and-screens plan	
Using only one screen at a time and turning off media that isn't being used			Avoiding social media use before school	
			Silencing phones by putting them on "do not disturb" during family time and playtime	

## Conclusion

While there are considerable concerns regarding the negative impacts of social media on adolescents, it also serves as a vital resource for adolescents and young adults. Much of the current research conducted on social media use has relied on focus groups and qualitative interviews, which can be vulnerable to reporting bias. Observational studies and objective tracking of social media may be needed to better understand social media use. Research has shown that social media engagement varies by race, gender, and other social factors, and future studies should explore how these demographic differences impact the positive outcomes associated with social media. Overall, social media can be an important tool for adolescents to find social connection, seek social support, and gain access to health information. Physicians can adopt a nuanced approach to addressing adolescent social media use.

## Data Availability

No datasets were generated or analysed during the current study.
